# GenUI: interactive and extensible open source software platform for de novo molecular generation and cheminformatics

**DOI:** 10.1186/s13321-021-00550-y

**Published:** 2021-09-25

**Authors:** M. Sicho, X. Liu, D. Svozil, G. J. P. van Westen

**Affiliations:** 1 CZ-OPENSCREEN: National Infrastructure for Chemical Biology, Department of Informatics and Chemistry, Faculty of Chemical Technology, University of Chemistry and Technology Prague, Technická 5, 166 28 Prague, Czech Republic; 2 CZ-OPENSCREEN: National Infrastructure for Chemical Biology, Institute of Molecular Genetics of the ASCR, v. v. i., Vídeňská 1083, 142 20 Prague 4, Czech Republic; 3 Computational Drug Discovery, Drug Discovery and Safety, Leiden Academic Centre for Drug Research, Einsteinweg 55, Leiden, The Netherlands

**Keywords:** Graphical user interface, De novo drug design, Molecule generation, Deep learning, Web application

## Abstract

Many contemporary cheminformatics methods, including computer-aided de novo drug design, hold promise to significantly accelerate and reduce the cost of drug discovery. Thanks to this attractive outlook, the field has thrived and in the past few years has seen an especially significant growth, mainly due to the emergence of novel methods based on deep neural networks. This growth is also apparent in the development of novel de novo drug design methods with many new generative algorithms now available. However, widespread adoption of new generative techniques in the fields like medicinal chemistry or chemical biology is still lagging behind the most recent developments. Upon taking a closer look, this fact is not surprising since in order to successfully integrate the most recent de novo drug design methods in existing processes and pipelines, a close collaboration between diverse groups of experimental and theoretical scientists needs to be established. Therefore, to accelerate the adoption of both modern and traditional de novo molecular generators, we developed Generator User Interface (GenUI), a software platform that makes it possible to integrate molecular generators within a feature-rich graphical user interface that is easy to use by experts of diverse backgrounds. GenUI is implemented as a web service and its interfaces offer access to cheminformatics tools for data preprocessing, model building, molecule generation, and interactive chemical space visualization. Moreover, the platform is easy to extend with customizable frontend React.js components and backend Python extensions. GenUI is open source and a recently developed de novo molecular generator, DrugEx, was integrated as a proof of principle. In this work, we present the architecture and implementation details of GenUI and discuss how it can facilitate collaboration in the disparate communities interested in de novo molecular generation and computer-aided drug discovery.

## Introduction

Due to significant technological advances in the past decades, the body of knowledge on the effects and roles of small molecules in living organisms has grown tremendously [1, 2]. At present, we assume the number of entries across all databases to be in the range of hundreds of millions or billions (10^8^–10^9^) [3–5] and a large portion of this data has also accumulated in public databases such as ChEMBL [6, 7] or PubChem BioAssay [1]. However, the size of contemporary databases is still rather small when compared to some estimates of the theoretical size of the drug-like chemical space, which may contain up to 10^33^ unique structures according to a recent study [8]. However, it should be noted that numerous studies in the past reported numbers both bigger and smaller depending on the definition used [8–11]. In addition, considering that only 1–2 measured biological activities per compound are available [12], the characterization of known compounds also needs to be expanded.

For a long time, de novo drug design algorithms for systematic and rational exploration of chemical space [13–15] and quantitative structure–activity relationship (QSAR) modeling [16] have been considered promising and useful cheminformatics tools to efficiently broaden our horizons with less experimental costs and without the need to exhaustively evaluate as many as 10^33^ possible drug-like compounds to find the few of interest. The relevance of QSAR modeling and de novo compound design for drug discovery has been discussed many times [13–21], but these approaches can be just as useful in other research areas [16]. In chemical biology, new tool compounds and chemical probes can be discovered with these methods as well [22].

**Fig. 1:**
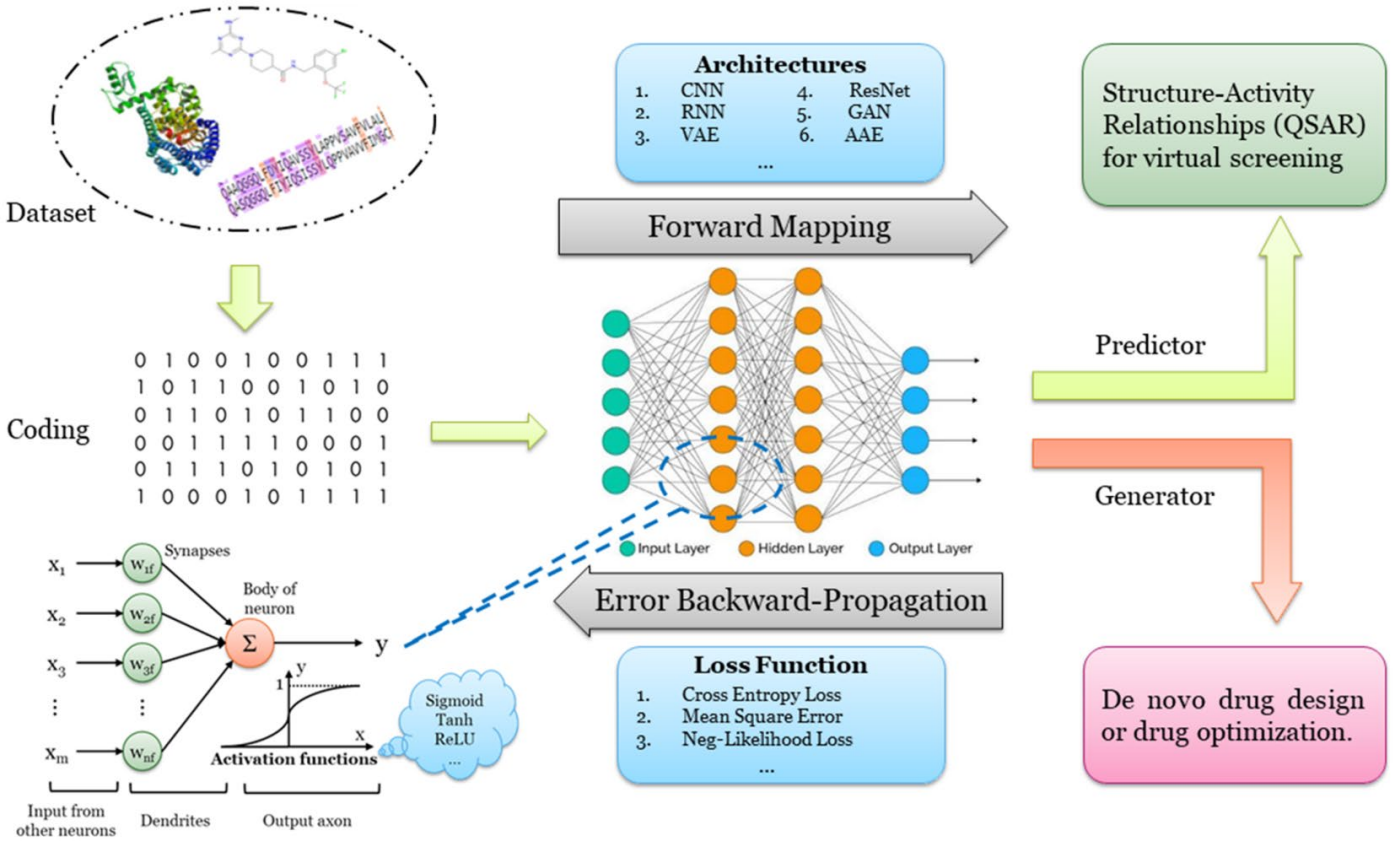
Schematic view of a typical cheminformatics workflow involving a DNN. First, a data set of compound structures and their measured activities on the desired target molecule (most often a protein) is compiled and encoded to suitable representation. Second, the encoded data is used as input of the neural network in forward mapping. A large number of architectures can be used with recurrent neural networks (RNNs) and convolutional neural networks (CNNs) as the most popular examples. Finally, the neural network is trained by backpropagating the error of a suitable loss function to adjust the activations inside the network so that the loss is minimized. Depending on the architecture, the network is trained either as a bioactivity predictor (e.g. a QSAR model) or as a molecular generator

Thanks to the rapid growth of bioactivity databases and widespread utilization of graphical processing units (GPUs) the efforts to develop powerful data-driven approaches for de novo compound generation and QSAR modeling based on deep neural networks (DNNs) has grown substantially (Fig. 1) [19]. The most attractive feature of DNNs for de novo drug design is their ability to probabilistically generate compound structures [13, 23]. DNNs are able to take non-trivial structure–activity patterns into account, thereby increasing the potential for scaffold hopping and the diversity of designed molecules [24, 25]. A number of generators based on DNNs was developed recently demonstrating the ability of various network architectures to generate compounds of given properties [13, 23, 26–29]. However, it should also be noted that the field of de novo drug design and molecular generation also has a long history of evolutionary heuristic methods with genetic algorithms on the forefront [20]. These traditional methods are still being investigated and developed [30–35] and it is yet to be established how they compare to the novel DNN-based approaches [13].

Although de novo molecular design algorithms have been in development for multiple decades [36] and experimentally validated active compounds have been proposed [18, 37–44], these success stories are still far away from the envisaged performance of the ‘robot scientist’ [45–47]. Successful development of a completely automated and sufficiently accurate and efficient closed loop process has been elusive, but significant advances have been made nonetheless [48]. However, even with encouraging results suggesting that full automation of the drug discovery process might be possible [18, 49–51], human insight and manual labor are still necessary to further refine and evaluate the compounds generated by de novo drug design algorithms. In particular, human intervention is of utmost importance in the process of compound scoring whereby best candidates are prioritized for synthesis and experimental validation [18, 51]. In this instance, the contributions of artificial intelligence (AI) are significant and AI algorithms can work independently to some extent, but expert knowledge is still important to interpret and refine such results and the creation of comprehensive graphical user interfaces (GUIs) and interoperable software packages can facilitate more direct involvement of experts from various fields.

Though many in silico compound generation and optimization tools are available for free [52], it is still an exception that these approaches are routinely used since the vast majority of methods described in the literature serve only as a proof of concept and can rarely be considered production-ready software. In particular, they lack a proper GUI through which non-experts could easily access the algorithms and analyze their inputs and outputs in a convenient way. While there are many notable exceptions [33, 35, 53, 54], the implemented GUIs are often simplistic and intended to be used only with one particular method. In addition, many molecular generators would also benefit from a comprehensive and easy to use application programming interface (API) that would enable easier integration with existing computational tools and infrastructures. Recently an open source tool called Flame was presented that offers many of the aforementioned features in the field of predictive QSAR modeling [55]. Such integrated frameworks from the realm of de novo compound generation are much more rare, however. To the best of our knowledge, BRADSHAW [56] and Chemistry42 [57] are the only two that were disclosed in literature recently and they unfortunately have not been made available as open source, which limits their use by the scientific community. On the other hand, it should be noted that there has been effort to develop open and interactive databases of generated structures as evidenced by the most recent example, cheML.io [58], which allows open access to the generated structures, but does not support “on-the-fly” generation. We argue that the lack of easy to use and auditable information systems for de novo drug design is a factor leading to some level of disconnection between medicinal and computational chemists [59], which can stand in the way of effective utilization of many promising de novo drug design tools.

Therefore, in this work we present the development of GenUI, a cheminformatics software framework that provides a GUI and APIs for easy use of molecular generators by human experts as well as their integration with existing drug discovery pipelines and other automated processes. The GenUI framework integrates solutions for import, generation, storage and retrieval of compounds, visualization of the created molecular data sets and basic utilities for QSAR modeling. Therefore, it is also suitable for many basic cheminformatics tasks (i.e. visualization of chemical data sets or simple QSAR modeling).

All GenUI features can be easily accessed through the web-based GUI or the Representational State Transfer API (REST API) to ensure that both human users and automated processes can interact with the application with ease. Integration of new molecular generators and other features is facilitated by a documented Python API while quick GUI customization is possible with an extensive library of components implemented with the React.js JavaScript library. To demonstrate the features of the GenUI framework, our recently published molecular generator DrugEx [60] was integrated within the GenUI ecosystem. The source code of the GenUI platform is distributed under the MIT open-source license [61–63] and several Docker [64–66] images are also available online for quick deployment [67].

## Implementation

### Software architecture

**Fig. 2:**
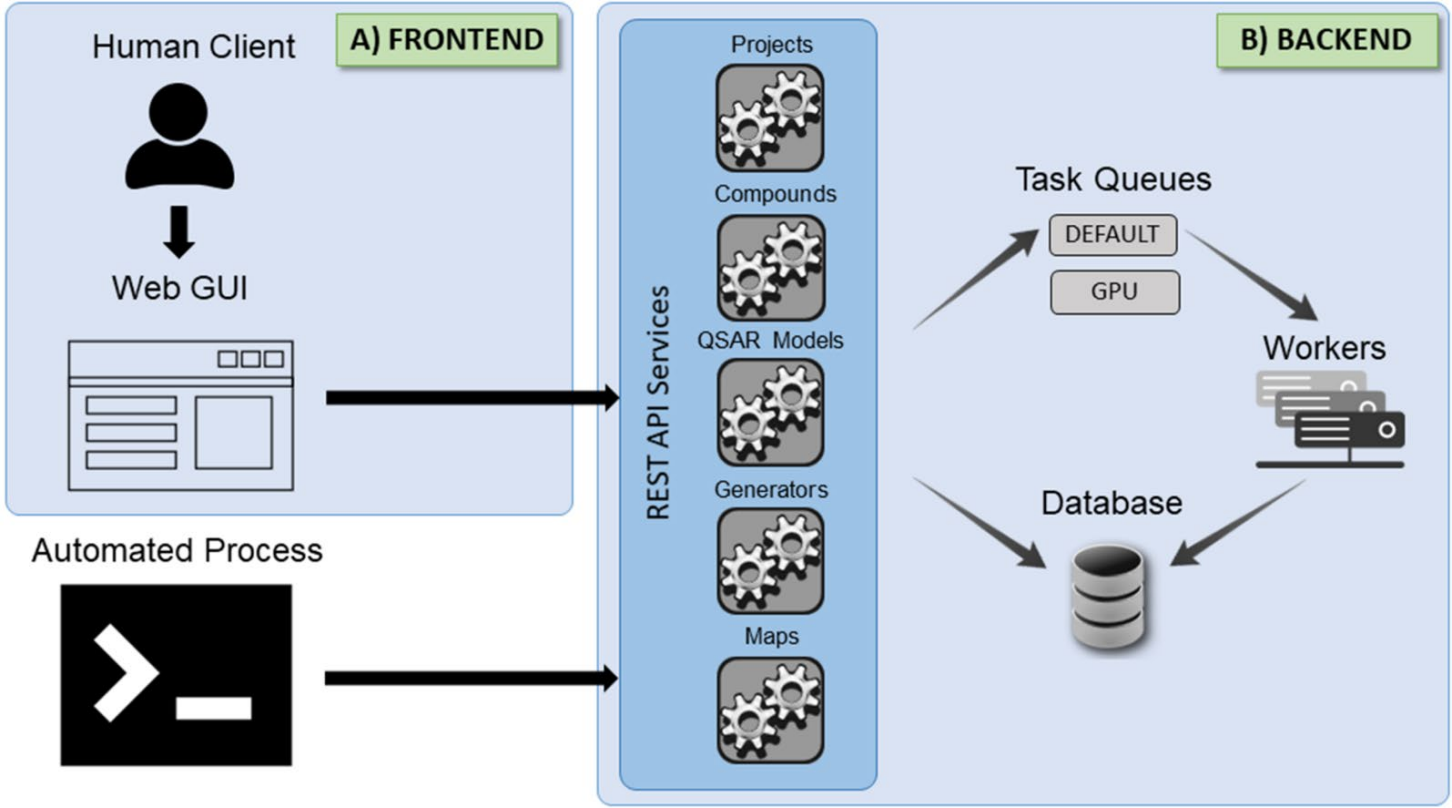
Schematic depiction of the GenUI platform architecture. On the frontend (**A**), users interact with the web-based GUI to access the backend server REST API services (**B**). All actions and data exchange are facilitated through REST API calls so that any automated process can also interact with GenUI. The backend application comprises five REST API services each of which has access to the data storage and task queue subsystems. The services can issue computationally intensive and long-running asynchronous tasks to backend workers to ensure sufficient responsiveness and scalability. In the current implementation, tasks can be submitted to two queues: (1) the default CPU queue, which handles all tasks by default, or (2) the GPU queue, intended for tasks that can be accelerated by the use of GPUs

User interaction with GenUI happens through the frontend web client that issues REST API calls to the backend, which comprises five services (Fig. 2). However, advanced users may also implement clients and automated processes that use the REST API directly.

The five backend services form the core parts of GenUI and can be described as follows:

1. “Projects” service handles user account management, authorization, and workflows. It is used to log in users and organize their work into projects.
2. “Compounds” service manages the compound database including deposition, standardization, and retrieval of molecules and the associated data (i.e. bioactivities, physicochemical properties, or chemical identifiers).
3. “QSAR models” service facilitates the training and use of QSAR models. They can be used to predict biological activities of the generated compounds, but they are also integral to training of many molecular generators.
4. “Generators” service is responsible for the integration of de novo molecular generators. It is meant to be used to set up and train generative algorithms whether they are based on traditional approaches or deep learning.
5. “Maps” service enables the creation of 2D chemical space visualizations and integration of dimensionality reduction algorithms.

In the following sections, the design and implementation of each part of the GenUI platform will be described in more detail.

### Frontend

#### Graphical user interface (GUI)

The GUI is implemented as a JavaScript application built on top of the React.js [68] web framework. The majority of graphical components is provided by the Vibe Dashboard open-source project [69], but the original collection of Vibe components was considerably expanded with custom components to fetch, send, and display data exchanged with the GenUI backend. In addition, frameworks Plotly.js [70], Charts.js [71] and ChemSpace.js [72] are used to provide helpful interactive figures.

**Fig. 3:**
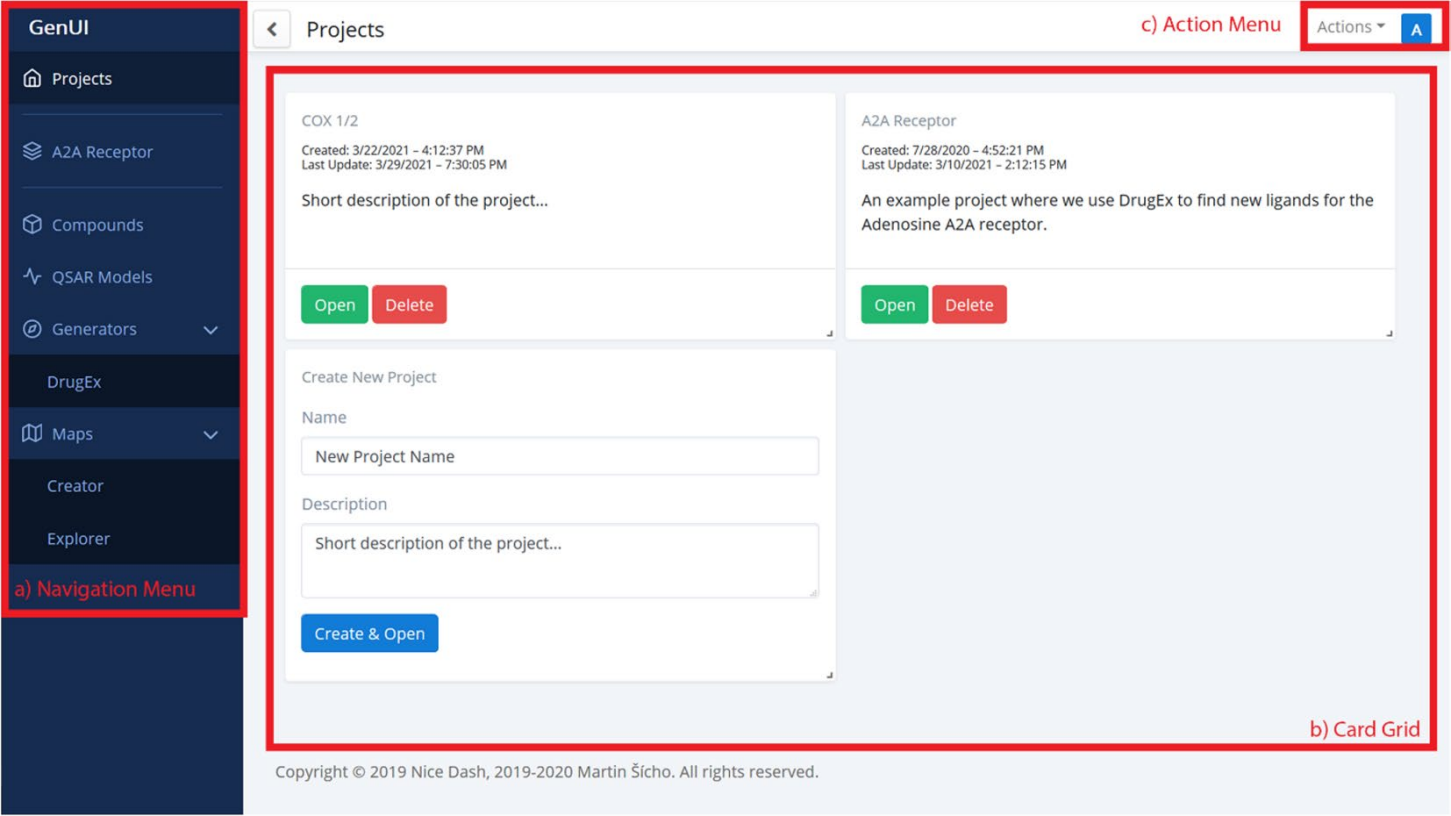
A screenshot showing part of the GenUI web GUI. In the figure, the GUI is in a state where the “A2A Receptor” project is already open and the navigation menu on the left can be used to access its data. The GUI consists of three main parts: (a) navigation menu, (b) card grid and (c) action menu. The navigation menu is used to browse data associated with various GenUI services (“Projects” in this case). If a link is clicked in the navigation menu, the data of the selected service is displayed as a grid of interactive cards. Each card allows the users to manage particular data items (a project in this case). The action menu in the top right is also updated depending on the service selected in the navigation menu and performs actions not related to a particular data item. In this case, the action menu was used to bring up the project creation form represented by the card in the bottom left of the card grid

The GUI reflects the structure of the GenUI backend services (Figs. 2 and 3). Each backend service (“Projects”, “Compounds”, “QSAR models”, “Generators”, and “Maps”) is represented as a separate item in the navigation menu on the left side of the interface (Fig. 3a). Upon clicking a menu item, the corresponding page opens rendering a grid of cards (Fig. 3b) that displays the objects corresponding to the selected backend service. Various actions related to the particular service can be performed from the action menu in the top right of the interface (Fig. 3c).

##### Projects

The “Projects” interface serves as a simple way to organize user workflows. For example, a project can encapsulate a workflow for the generation of novel ligands for one protein target (Fig. 3). Each project contains imported compounds, QSAR models, molecular generators and chemical space maps. The number of projects per user is not limited and they can be deleted or created as needed.

##### Compounds

**Fig. 4:**
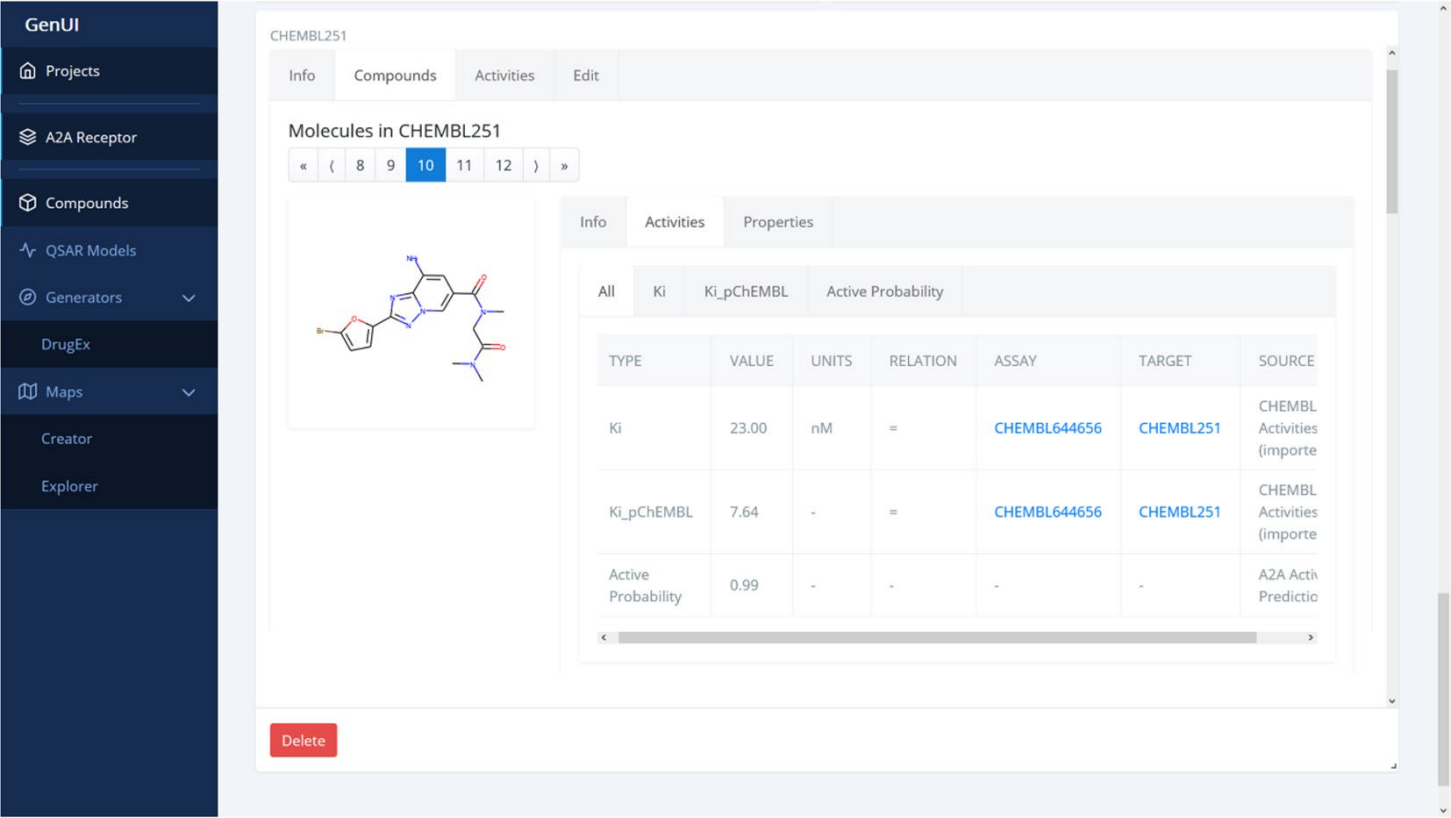
A screenshot showing part of the “Compounds” GUI. On this page, users can import data sets from various sources. A card representing an already imported data set from the ChEMBL database [7] is shown. The position and size of each displayed card can be modified by either dragging the card (reposition) or adjusting the bottom right corner (size change). The card shown is currently expanded over two rows of the card grid (Fig. 3b) in order to accommodate the displayed data better. The “Activities” tab in the compound overview shows summary of the biological activity data associated with the compound. The activities are grouped by type and aside from experimentally determined activities the interface also displays activity predictions of available QSAR models. For example, in the view shown the “Active Probability” activity type is used to denote the output probability from a classification QSAR model. Each activity value also contains information about its origin (the “Source” column) so that it can be tracked back to its source

Each project may contain any number of compound sets (Fig. 4). Each set of compounds can have a different purpose in the project and come from a different source. Therefore, the contents of each card on the card grid depend on the type of compound set the card represents. Compounds can be generated by generators, but also imported from SDF files, CSV files or obtained directly from the ChEMBL database [6, 7]. New import filters can be easily added by extending the Python backend and customizing the components of the React API accordingly (see “14” and “10”). For each compound in the compound set the interface can display its 2D representation (Fig. 4), molecular identifiers (i.e. SMILES, InChI, and InChIKey), reported and predicted activities (Fig. 4) and physicochemical properties (i.e. molecular weight, number of heavy atoms, number of aromatic rings, hydrogen bond donors, hydrogen bond acceptors, logP and topological polar surface area).

##### QSAR models

**Fig. 5:**
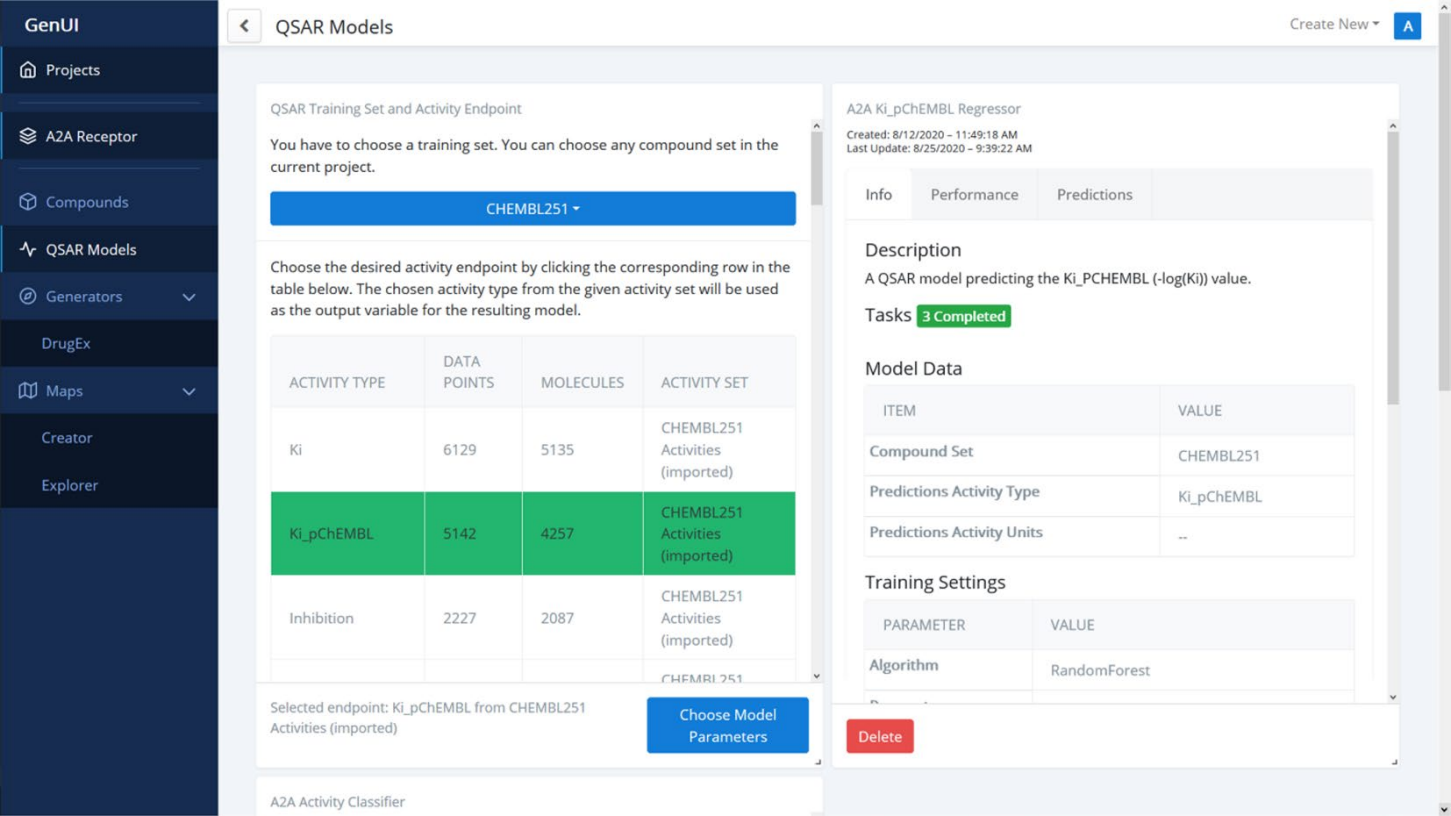
A screenshot showing part of the “QSAR Models” GUI. The card on the left side of the screen shows how training data is chosen for a new model while the card on the right shows metadata about an already trained model

**Fig. 6:**
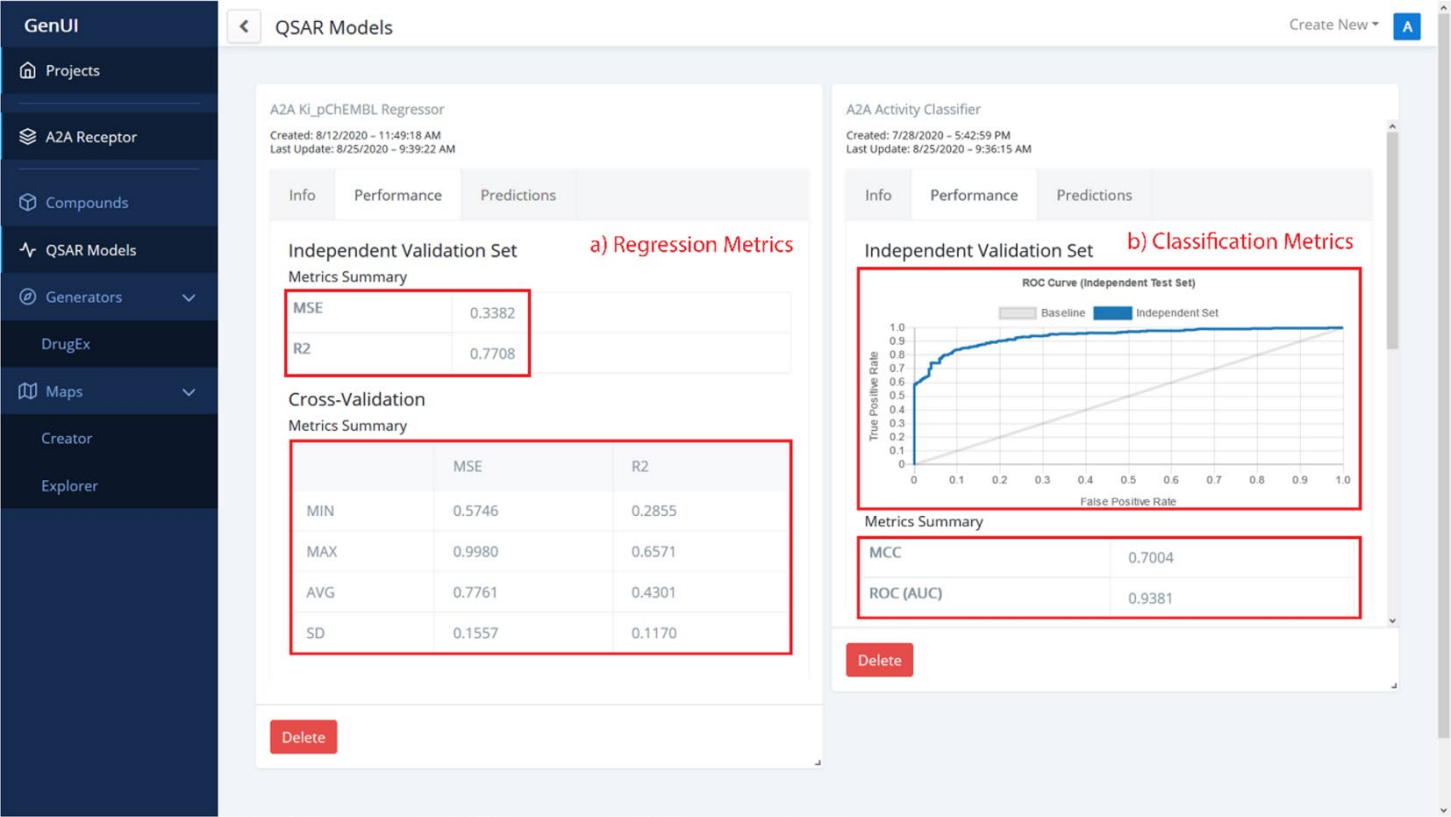
Performance evaluation view for (a) regression and (b) classification QSAR model. In (a), the mean-squared error (MSE) and the coefficient of determination (R2) are used as validation metrics. In (b), the performance is measured on a hold out independent validation test set with the Matthews correlation coefficient (MCC) and the area under the receiver operating characteristic (ROC) curve (AUC). The ROC curve itself is also displayed above the metrics

All QSAR models trained or imported in the given project are available from the “QSAR Models” page (Figs. 5, 6). Each QSAR model is represented by a card with several tabs. The “Info” tab contains model metadata, as well as a serialized model file to download (Fig. 5). The “Performance” tab lists various performance measures of the QSAR model obtained by cross-validation or on an independent hold out test set (Fig. 6). The validation procedure can be adjusted by the user during model creation (Fig. 5). Making predictions with the model is possible under the “Predictions” tab. Each QSAR model can be used to make predictions for any compound set listed on the “Compounds” page and the calculated predictions will then become visible in that interface as well (Fig. 4).

New QSAR models are submitted for training with a creation card (Fig. 5) that helps users choose model hyperparameters and a suitable training strategy (i.e. the characteristics of the independent hold out validation set, the number of cross-validation folds or the choice of validation metrics). The “Info” tab of a trained model contains important metadata as well as a hyperlink to export the model and save it as a reusable Python object. This import/export feature enables users to archive and share their work, enhancing the reusability and reproducibility of the developed models [73]. The “Performance” tab can be used to observe model performance data according to the chosen validation scheme (Fig. 6). This information is different depending on the chosen model type (regression vs. classification, Fig. 6a vs. b) and the parameters used (i.e. the choice of validation metrics). Additional performance measures and machine learning algorithms can be integrated with the backend Python API. Creation of such extensions does not even require editing of the GUI for many standard algorithms (see “14”).

##### Generators

**Fig. 7:**
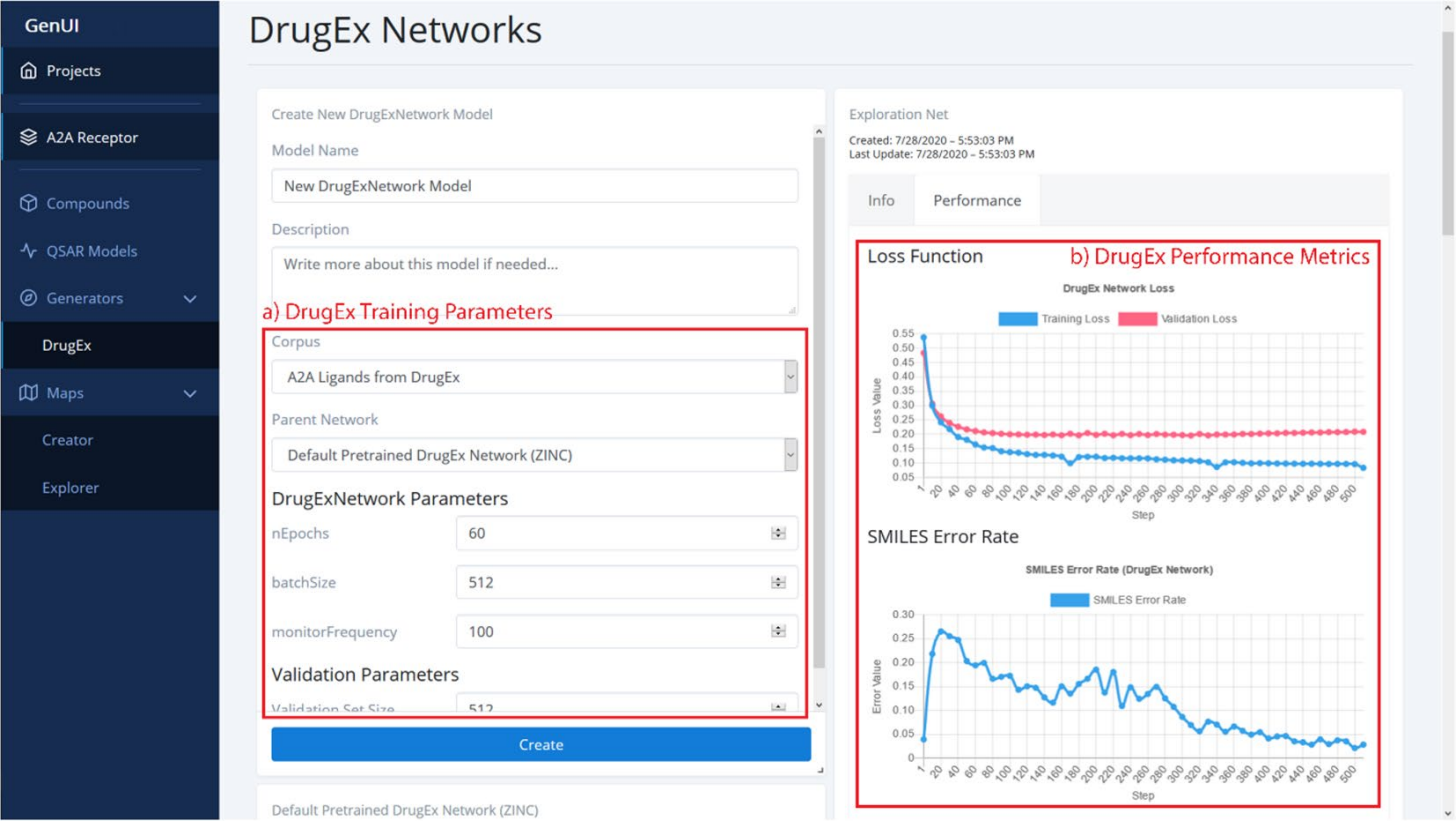
A screenshot showing part of the “DrugEx” GUI with a model creation card with (a) DrugEx training parameters and (b) performance overview of a trained DrugEx network. In (a) the fields to define the compound set for the process of fine-tuning the exploitation (‘parent’) recurrent neural network trained on the ZINC data set [60] are shown. In addition, the form provides fields to set the number of learning epochs, training batch size, frequency of performance monitoring and size of the validation set. In (b) the “Performance” tab tracks model performance. It shows values of the loss function on the training set and validation set (top) and the SMILES error rate (bottom) at each specified step of the training process. The performance view is updated according to the chosen monitoring frequency in real time as the model is being trained. Each model also has the “Info” tab which holds the same information as for QSAR models

Under the “Generators” menu item, the users find a list of individual generators implemented in the GenUI framework (Fig. 7). Currently, only the DrugEx generator [60] is available, but other generators can be added by extending the Python backend (see “14”) and customizing the existing React components (see “10”). It is likely that some generators will have specific requirements on the GUI elements used on the page and, thus, the GUI is organized so that each new generator is integrated as a completely new page accessed from the navigation menu on the left. Many of the graphical elements used in the DrugEx extension (i.e. the model creation form, Fig. 7a) are simply customized elements from the library of GenUI graphical components. In fact, the GUI for DrugEx is based on the same React components as the “QSAR Models” view.

The DrugEx method consists of two networks, an exploitation network and an exploration network, that are trained together [60]. The exploration network is used to fine-tune the exploitation network, which is then trained under the reinforcement learning framework to optimize the agent that generates the desired compounds. Therefore, the interface of DrugEx was divided into two parts: (1) for training DrugEx exploration networks (Fig. 7) and (2) for training DrugEx agents (not shown). In this case, the graphical elements needed for the two types of networks are very similar and are just placed as two card grids under each other. The only custom React components made for this interface are the figures used to track real time model performance (Fig. 7b). All other components come from the original GenUI React library (see “10”) and are simply configured to use data from the DrugEx extension REST API endpoints.

Like QSAR models, DrugEx networks can also be serialized and saved as files. For example, a cheminformatics researcher can build a DrugEx model outside of the GenUI ecosystem (i.e. using the scripts published with the original paper [60]) and provide the created model files to another researcher who can import and use the model from the GenUI web-based GUI. Therefore, it is easy to share work and accommodate various groups of users in this way.

##### Maps

**Fig. 8:**
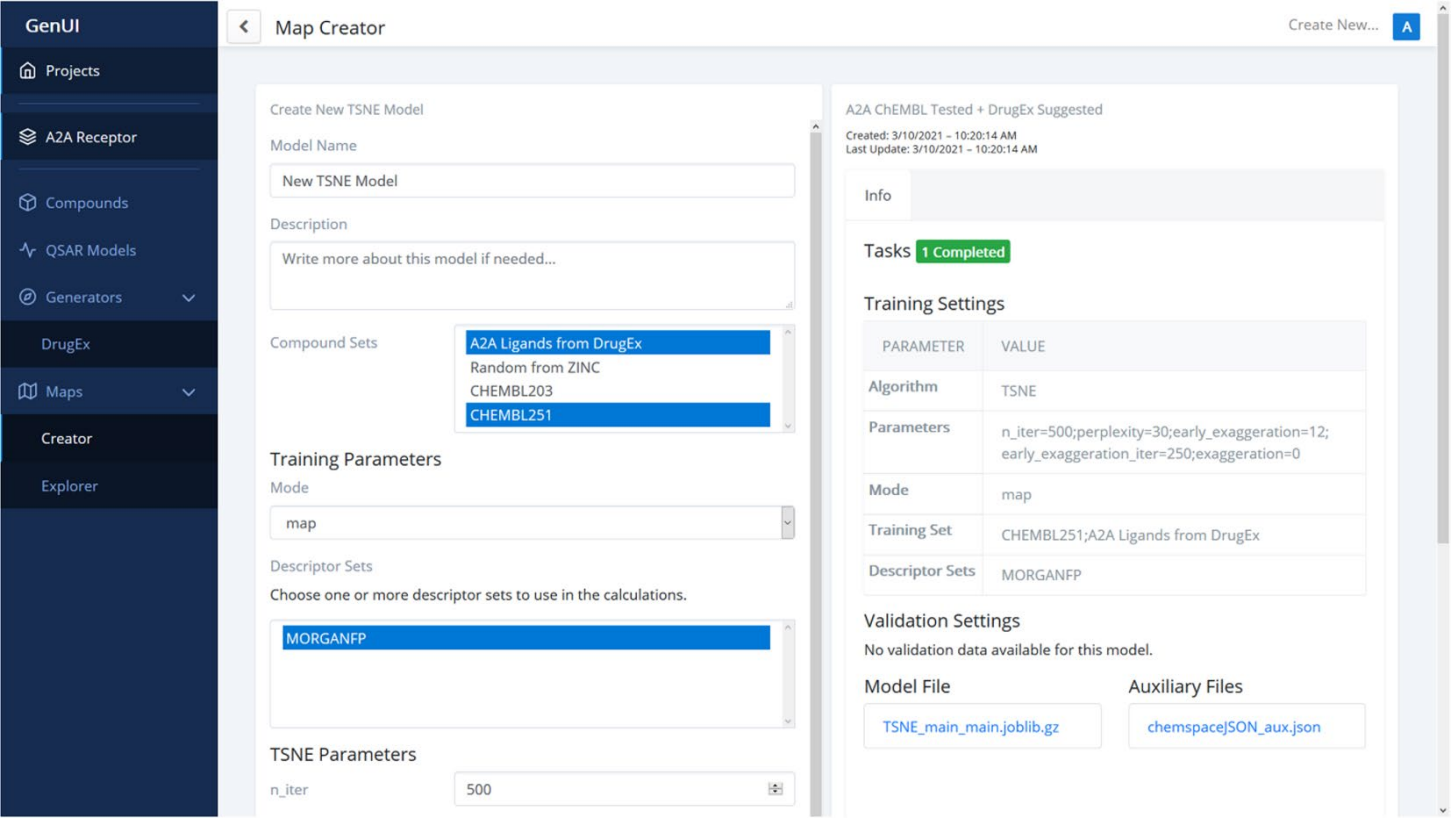
The “Creator” interface of GenUI “Maps” page. On the left a form to create a new t-SNE [74] mapping of two sets of compounds using Morgan fingerprints is shown while information about an existing map can be seen on the right

**Fig. 9:**
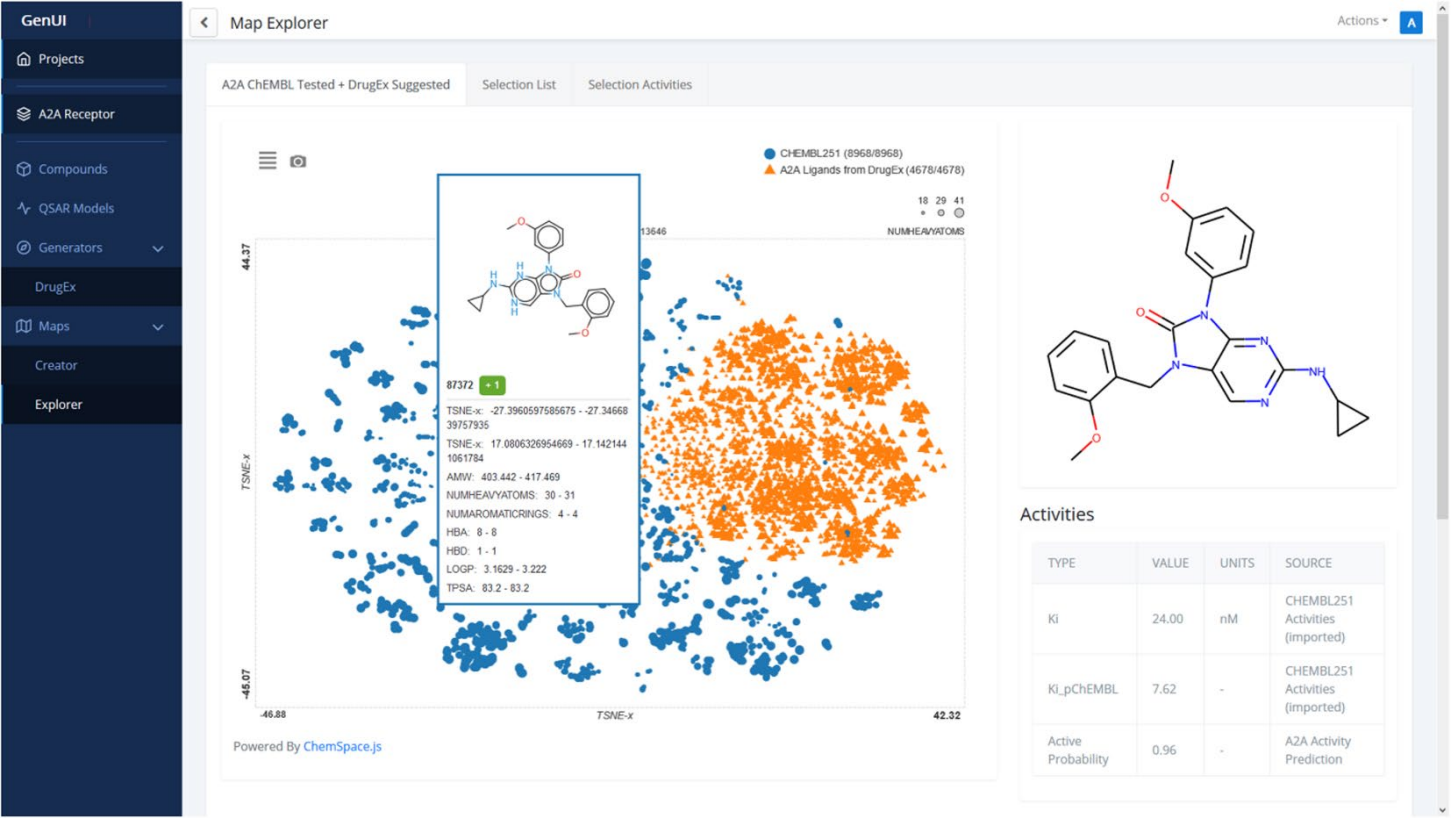
A screenshot showing the “Explorer” part of the “Maps” GUI. The interactive plot on the left side of the screen is provided by the ChemSpace.js library [72]. Each point in this visualization corresponds to one molecule. In this particular configuration, the shapes and colors of the points indicate the compound set to which the compounds belong to. The color scheme of points can be changed with the menu in the top left corner of the plot. It is possible to color points by biological activities, physicochemical properties and other data associated with the compounds. The same can also be done with the size of the points. The points drawn in the map are interactive and hovering over a point shows a box with information about the compound inside and on the right side of the map. Groups of points can also be selected by drawing a rectangle over them in which case a list of selected compounds is shown in the “Selection List” tab (Fig. 10) and their bioactivity data is summarized under the “Selection Activities” tab (Fig. 11)

Interactive visualization of chemical space is available under the “Maps” menu item. The menu separates the creation of the chemical space visualization, the “Creator” page (Fig. 8), and its exploration, the “Explorer” page (Fig. 9).

The “Creator” page is implemented as a grid of cards each of which represents an embedding of chemical compounds in 2D space (Fig. 8). Implicitly, the GenUI platform enables t-SNE [74] embedding (provided by openTSNE [75]). However, new projection methods can be easily added to the backend through the GenUI Python API with no need to modify the GUI (see “14”) [76].

**Fig. 10:**
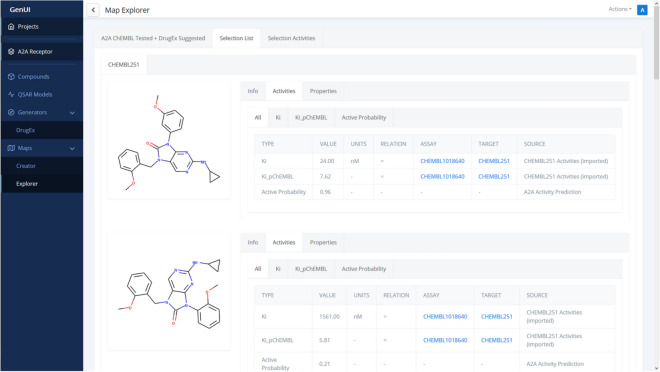
View of the “Selected List” tab of the “Explorer” page. The tab shows the selected molecules in the map as a list which is the same as the one used in the “Compounds” view (Fig. 4). For easier navigation, the compounds are also grouped by the compound set they belong to and the view for each set can be accessed by switching tabs above the displayed list (only one compound set, CHEMBL251, is present in this case)

**Fig. 11:**
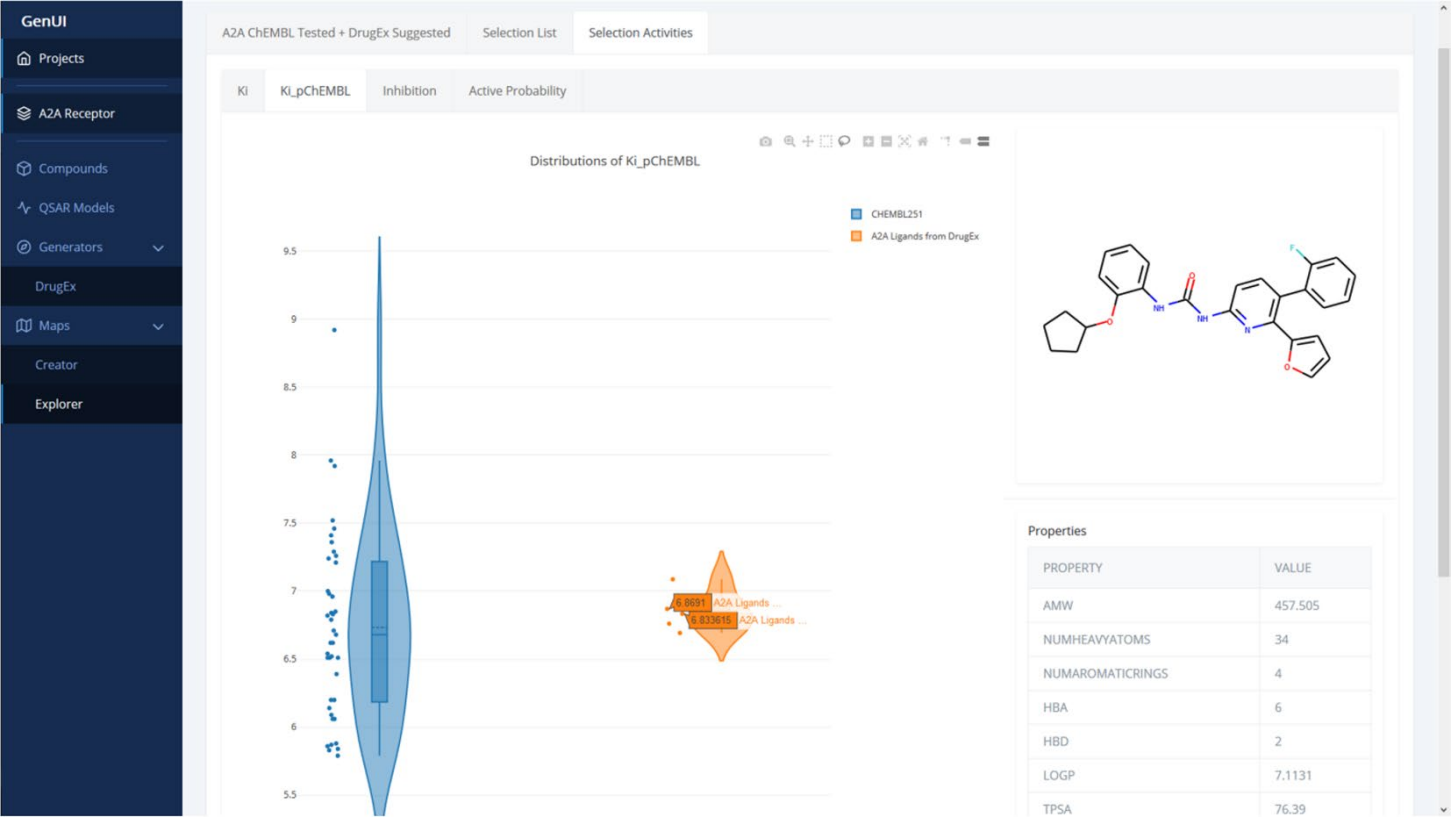
View of the “Selection Activities” tab of the “Explorer” page. In this view, violin plots representing distributions of activities in the set of selected compounds are displayed. Each violin plot corresponds to one compound set and one activity type. The violin plots are also interactive and hovering over points updates the compound structure and its physicochemical properties are displayed on the right

The purpose of the “Explorer” page (Fig. 9) is to interactively visualize chemical space embedding prepared in the “Creator”. In the created visualization, users can explore compound bioactivities, physicochemical properties, and other measurements for various representations and parts of chemical space. Thanks to ChemSpace.js [72] up to 5 dimensions can be shown in the map at the same time with various visualization methods: X and Y coordinates, point color, point size and point shape. The map can be zoomed in by drawing a rectangle over a group of points. Such points form a selection and their detailed information is displayed under the “Selected List” (Fig. 10) and “Selected Activities” tabs (Fig. 11).

#### JavaScript API

**Fig. 12:**
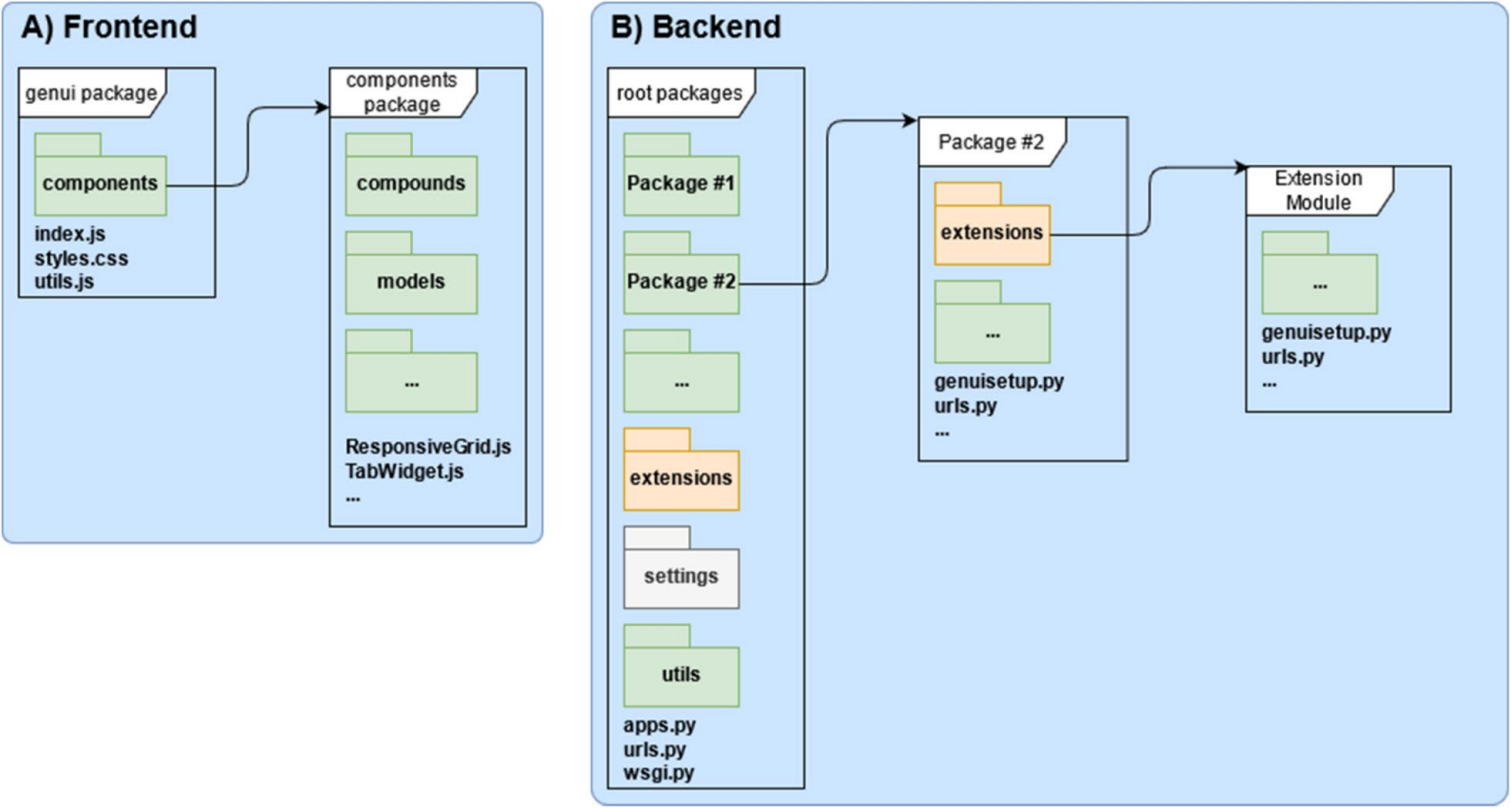
Organization of the GenUI frontend (**A**) and backend (**B**) packages. The frontend React library (**A**) contains customized styles, utility functions and the React components used in the GenUI web client. The React components are further divided into groups related to the structure of the GenUI interface. Schematic depiction of the GenUI backend Python code. The backend (**B**) is structured as a standard Django project (designated by its settings package and the urls and wsgi modules). The GenUI code itself is divided into a number of root packages that are further divided into subpackages. The extensions subpackage is specific to GenUI and is used to automatically discover and configure extension modules. GenUI extensions and packages typically define the genuisetup module, which is used to configure the extension when the Django project is run

Two main considerations in the development of GenUI are reusability and extensibility. Therefore, the frontend GUI comprises a large library of over 50 React components that are encapsulated in a standalone package (Fig. 12A). The package is organized into subpackages that follow the structure and hierarchy of design elements in the GenUI interface. In the following sections, we use the two most important groups of the React API components as case studies to illustrate how the frontend GUI can be extended. The presented components are “Model Components”, used to add new trainable models, and “REST API Components”, used to fetch and send data between the frontend and the GenUI REST API.

##### Model components

**Fig. 13:**
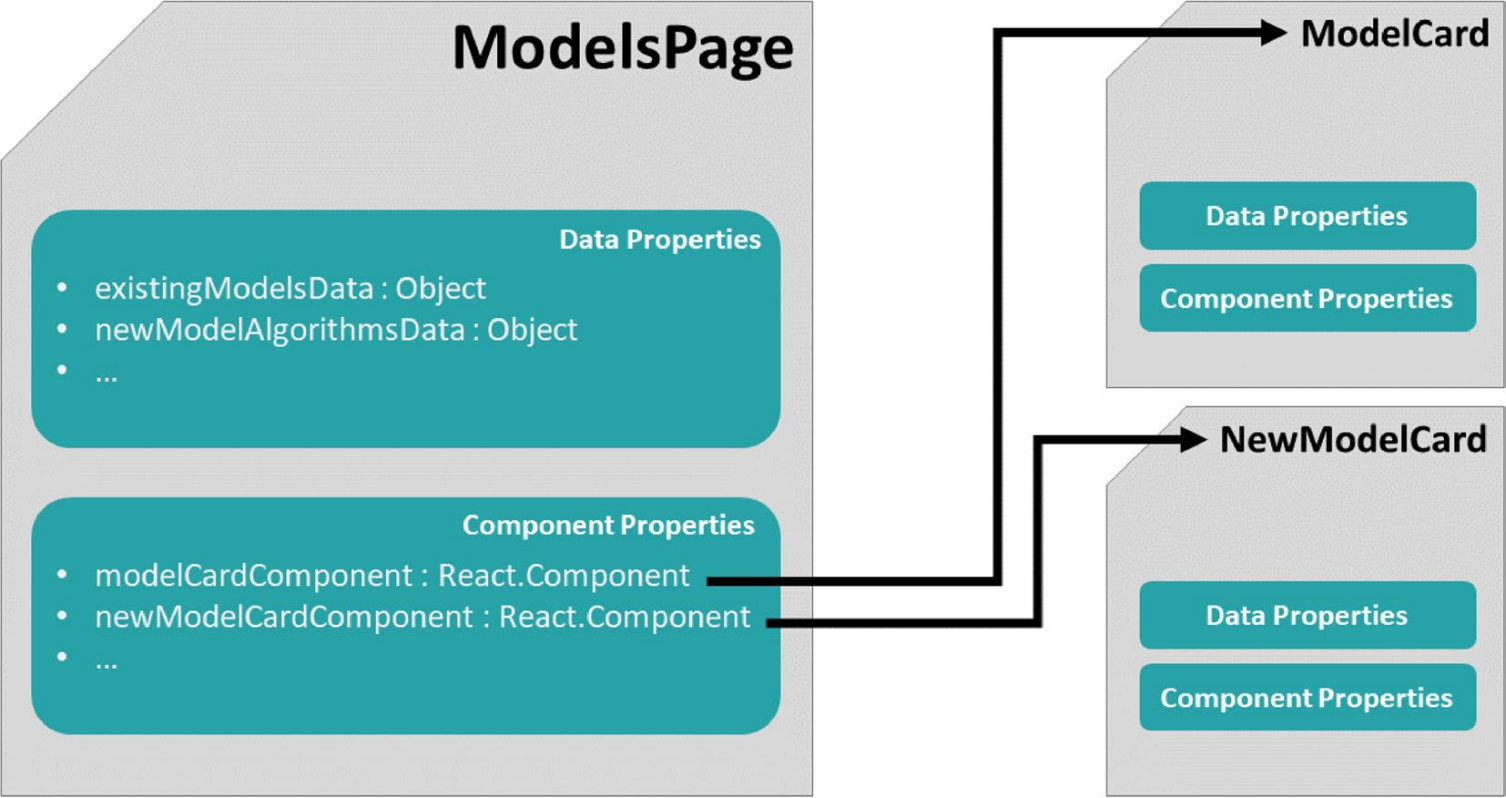
A simplified illustration of the high-level components in the GenUI React API for rendering model cards. The main *ModelsPage* component has two kinds of attributes (called “properties” in React): (a) *data properties* and (b) *component properties*. The values of data properties are used to display model data while the values of component properties are used as child components and injected into the GUI at appropriate places. If no component property is specified, default components are used as children instead (i.e. *ModelCard* and *NewModelCard*). The child components can accept data and component properties as well from their parent (i.e. *ModelsPage*). This creates a hierarchy of reusable components that can be easily assembled and configured to accommodate the different needs of each model view in a standardized and consistent manner

Much of the functionality of the GenUI platform is based on trained models. The “QSAR Models”, “DrugEx” and “Maps” pages all borrow from the same library of reusable GenUI React components (Fig. 12A). At the core of the “models” component library (Fig. 12A) is the *ModelsPage* component (Fig. 13). *ModelsPage* manages the layout and data displayed in model cards. When the users select to build a new model, the *ModelsPage* component is also responsible to show a card with the model creation form. The information that the *ModelsPage* displays can be customized through various React properties (Fig. 13) that represent either data (data properties) or other components (component properties). Such an encapsulation approach and top-down data flow is one of the main strengths of the React framework. This design is very robust since it fosters appropriate separation of concerns by their encapsulation inside more and more specialized components. This makes the code easy to reuse and maintain.

##### REST API components

Because the GUI often needs to fetch data from the backend server, several React components were defined for that purpose. In order to use them, one just needs to provide the required REST API URLs as React component properties. For example, the *ComponentWithResources* component configured with the ‘/maps/algorithms/’ URL will get all available embedding methods as JSON (JavaScript Object Notation) and converts the result to a JavaScript object. Many components can also periodically update the fetched data, which is useful for tracking information in real time. For paginated data there is also the *ApiResourcePaginator* component that only fetches a new page if a given event is fired (i.e. user presses a button). This makes it convenient to create efficient GUIs for larger data sets. In addition, user credentials are also handed over to the server automatically in all of these components.

Many more specialized components are also available to fetch specific information. For example, the *TaskAwareComponent* tracks URLs associated with background asynchronous tasks and it regularly passes information about completed, running, or failed tasks to its child components. However, other specialized components exist that automatically fetch and format pictures of molecules, bioactivities, physicochemical properties or create, update and delete objects in the UI and the server [62].

### Backend

The backend services are the core of the GenUI platform and the GenUI Python API provides a convenient way to write backend extensions (i.e. add new molecular generators, compound import filters, QSAR modeling algorithms, and dimensionality reduction methods for chemical space maps). All five backend services (Fig. 2) are implemented with the Django web framework [77] and Django REST Framework [78]. For data storage, a freely available Docker [66] image developed by Informatics Matters Ltd. [79] is used. The Docker image contains an instance of the PostgreSQL database system with integrated database cartridge from the RDKit cheminformatics framework [80]. The integration of RDKit with the Django web framework is handled with the Django RDKit library [81]. All compounds imported in the database are automatically standardized with the current version of the ChEMBL structure curation pipeline [82].

Because the backend services also handle processing of long-running and computationally intensive tasks, the framework uses Celery distributed task queue [83] with Redis as a message broker [84] to dispatch them to workers. Celery workers are processes running in the background that consume tasks from the task queue and process them asynchronously. Workers can either run on the same machine as the backend services or they can be distributed over an infrastructure of computers (see “24”).

#### Python API

Django is a web framework that utilizes the Model View Template (MVT) design pattern to handle web requests and draw web pages. MVT is similar to the well-known Model View Controller (MVC) design pattern, but without a dedicated controller that determines what view needs to be called in response to a request. In MVT, the framework itself plays the role of the controller and makes sure that the correct view is called upon receiving a web request. In Django, the view is represented by a Python function or a method that returns various data types based on the nature of the request. The view can also take advantage of the Django templating engine to dynamically generate HTML pages. In both MVC and MVT, the model plays a role of a data access layer. The model represents the tables in the database and facilitates search and other data operations. GenUI does not use the Django templating engine, but rather handles all requests via REST API endpoints that manipulate data in JSON. This makes the frontend React application completely decoupled from the backend and also enables other clients to access the GenUI data in a convenient way by design (Fig. 2).

The GenUI backend codebase [63] follows the standard structure of any Django project and is divided into multiple Python packages that each encapsulate smaller self-contained parts (Fig. 12B). In GenUI, any package that resides in the root directory is referred to as the *root package*. Root packages facilitate many of the REST API endpoints (Fig. 2), but they also contain reusable classes that are intended to be built upon by extensions (see “17”, for example). In the following sections, some important features of the backend Python API are briefly highlighted. However, a much more detailed description with code examples is available on the documentation page of the project [76].

##### Extensions

Django is known for its strong focus on modularity and extensibility and GenUI tries to follow in its footsteps and support a flexible system of pluggable applications. Each of the GenUI root packages contains a Python package called *extensions* (Fig. 12B). The *extensions* package can contain any number of Django applications or Python modules, which ensures that the extending components of the GenUI framework are well-organized and loosely coupled.

Provided that GenUI extensions are structured a certain way they can take advantage of automatic configuration and integration (see “16”). Before the Django project is deployed, GenUI applications and extensions are detected and configured with the *genuisetup* command, which makes sure that the associated REST API endpoints are exposed under the correct URLs. The *genuisetup* command is executed with the *manage.py* script (a utility script provided by the Django library).

##### Automatic code discovery

The root packages of the GenUI backend library define many abstract and generic base classes to implement and reuse in extensions. These classes either implement the REST API or define code to be run on the worker nodes inside Celery tasks. Automatic code discovery uses several introspection functions and methods to find the derived classes of the base classes found in the root packages. By default, this is done when the *genuisetup* command is executed (see “15”).

For example, if the derived class defines a new machine learning algorithm to be used in QSAR modelling, automatic code discovery utilities make sure that the new algorithm appears as a choice in the QSAR modelling REST API and that proper parameter values are collected via the endpoint to create the model. Moreover, all changes also get automatically propagated to the web-based GUI because it uses the REST API to obtain algorithm choices for the model creation form. Thus, no JavaScript code has to be written to integrate a new machine learning algorithm. These concepts are also used when adding molecular generators, dimensionality reduction methods, or molecular descriptors.

##### Generic views and viewsets

When developing REST API services with the Django REST Framework [78], a common practice is to use generic views and sets of views (called viewsets). In Django applications, views are functions or classes that handle incoming HTTP requests. Viewsets are classes defined by the Django REST Framework that bring functionality of several views (such as creation, update or deletion of objects) into one single class. Generic views and viewsets are classes that usually do not stand on their own, but are designed to be further extended and customized.

The GenUI Python library embraces this philosophy and many REST API endpoints are encapsulated in generic views or viewsets. This ensures that the functionality can be reused and that no code needs to be written twice, as stated by the well-known DRY (“Don’t Repeat Yourself”) principle [85]. An example of such a generic approach is the *ModelViewSet* class that handles the endpoints for retrieval and training of machine learning models. This viewset is used by the *qsar* and *maps* applications, but also by the DrugEx extension. All these applications depend on some form of a machine learning model so they can take advantage of this interface, which automatically checks the validity of user inputs and sends model training jobs to the task queue.

##### Asynchronous tasks

Many of the GenUI backend services take advantage of asynchronous tasks which are functions executed in the background without blocking the main application. Moreover, tasks do not even have to be executed on the same machine as the caller of the task, which allows for a great deal of flexibility and scalability (see “24”).

The Celery task queue [83] makes creating asynchronous tasks as easy as defining a Python function [86]. In addition, some GenUI views already define their own tasks and no explicit task definition is needed in the derived views of the extensions. For example, the *compounds* root package defines a generic viewset that can be used to create and manage compound sets. The import and creation of compounds belonging to a new compound set is handled by implementing a separate initializer class, which is passed to the appropriate generic viewset class [76]. The initialization of a compound set can take a long time or may fail and, thus, should be executed asynchronously. Therefore, the viewset of the *compounds* application automatically executes the methods of the initializer class asynchronously with the help of an available Celery worker.

### Integration of new features with the two APIs

While a few examples of integrating new features to the GenUI platform have already been given for both Python and JavaScript, in this section a brief overview of all extensible features of the GenUI platform will be given. The vast majority of the features implemented in the reference platform presented in this work is realized through the extension system introduced earlier (see “15”). Extensions can use a wide selection of cheminformatics and data analysis tools each with their own level of complexity. Therefore, in this section we discuss the ease/difficulty of implementing the most common extensions and outline the problems the developers will face when developing each type of extension on both frontend and backend. All of the extensible use cases discussed here are also described in the project documentation with code examples [76].

#### Compounds import

Importing sets of compounds from various sources may require different approaches and as a result different kinds of interfaces. Therefore, the GenUI platform was designed with more flexibility in mind in this case. However, it also means that more configuration is needed from the developer. Extending the GenUI backend is accomplished by creating an extension application that defines the REST API URLs of the extension as well as views that will serve the defined URLs. GenUI provides a generic viewset class that can be derived from to make this process a matter of a few lines of code. The initializer class that handles the import itself also needs to be implemented by the developer of the extension, but an already prepared initializer base class is available in GenUI as well. Among other things, this base class also handles molecule standardization and clean up which ensures unified representation of chemical structures across data sets. In the frontend API, there is a selection of React components that can be used to build cards representing imported compound sets. The cards need very little configuration and automatically include metadata and the list of compounds in the compound set.

#### QSAR models

The backend model integration API is designed to provide easy and fast integration of simple machine learning algorithms even without the need to manually modify the frontend GUI. Adding a QSAR model can be as simple as adding a single class to the extension. The responsibility of this class is to use a machine learning algorithm to train and serialize a model upon receiving training data and predict unknown data from the deserialized model when requested. This class is also annotated with metadata about the model to be displayed in the frontend GUI. Therefore, in the simplest cases no URLs or customized GUI components need to be defined. The GenUI framework itself also performs cross-validation and independent set validation and data preprocessing. However, in many cases customized behavior, novel descriptor or validation metrics implementations might be necessary and in that case the developer may be required to define new URLs, views and modeling strategies. However, also in this case the GenUI platform attempts to make this process easier by providing generic viewsets and loosely coupled base class implementations that the developers can take advantage of. In addition, the interface to define molecular descriptors and validation metrics is designed with reusability in mind and also exposes the implemented features to other QSAR algorithms if needed.

#### Molecular generators

Molecular generators can be of various types and even those based purely on DNNs are often of different architectures and take advantage of diverse software frameworks. GenUI is designed in a fashion that is agnostic to the type of algorithm used and it leaves preprocessing of the training data (if any) and the generation of output solely on the developer of the extension. GenUI only defines the means to communicate data between the framework and the generator code. This also means that integration of a molecular generator requires more customization, the extent of which largely depends on the type of the generative algorithm used. The GenUI model integration API that is used for integration of QSAR models can also be used for integration of molecular generators based on DNNs and is used by the DrugEx extension. Therefore, integrating contemporary approaches that are mostly based on DNNs should be easier thanks to the possibility to follow the example of DrugEx as a proof of concept. Generators may also have different requirements on the information displayed in the GUI and, thus, it is expected that the GUI will be customized as well. However, if the generator takes advantage of the GenUI model integration API, this process is significantly simplified.

#### Chemical space maps

The dimensionality reduction methods used to create the chemical space maps shown in the GenUI interface are handled through the GenUI model integration API as well. Therefore, integration of these approaches is handled similarly to QSAR models and, thus, it comes with the same set of requirements and assumptions. Implementing a simple dimensionality reduction method will likely not require any steps beyond the definition of the one class that contains the implementing code and algorithm metadata.

### Deployment

#### Docker images

Since the GenUI platform consists of several components with many dependencies and spans multiple programming languages, it can be tedious to set up the whole project on a new system. Docker makes deployment of larger projects like this easier by encapsulating different parts of the deployment environment inside Docker images [64–66]. Docker images are simply downloaded and deployed on the target system without the need to install any other tools beside Docker. GenUI uses many official Docker images available on the Docker image sharing platform Docker Hub [87]. The PostgreSQL database with built-in RDKit cartridge [79], Redis [79, 88] and the NGINX web server [89, 90] are all obtained by this standard channel. In addition, we defined the following images to support the deployment of the GenUI platform itself [67]:

1. *genui-main*: Used to deploy both the frontend web application and the backend services.
2. *genui-worker*: Deploys a basic Celery worker without GPU support.
3. *genui-gpuworker*: Deploys a Celery worker with GPU support. It is the same as the *genui-worker*, but it has the NVIDIA CUDA Toolkit already installed.

The tools to build these images are freely available [67]. Therefore, developers can create images for extended versions of the GenUI that fit the needs of their organizations. In addition, the separation of the main application (*genui-main*) from workers also allows distributed deployment over multiple machines, which opens up the possibility to create a scalable architecture that can quickly accommodate teams of varying sizes.

### Future directions

Although the GenUI framework already implements much of the functionality needed to successfully integrate most molecular generators, there are still many aspects of the framework that can be improved and the framework is under continuous development. For instance, it would be beneficial if more sources of molecular structures and bioactivity information are integrated in the platform besides ChEMBL (i.e. PubChem [91], ZINC [92], DrugBank [93], BindingDB [94] or Probes and Drugs [95]). Currently, GenUI also lacks features to perform effective similarity and substructure searches, which we see as a crucial next step to improve the appeal of the platform to medicinal chemists. The current version of GenUI would also benefit from extending the sets of descriptors, QSAR machine learning algorithms and chemical space projections since the performance of different methods can vary across data sets. Finally, the question of synthesizability of the generated structures should also be addressed and a system for predicting chemical reactions and retrosynthetic pathways could also be very useful to medicinal chemists if integrated in the GUI (i.e. by facilitating connection to a service such as the IBM RXN [96] or PostEra Manifold [97]).

Even though it is hard to determine the requirements of every project where molecular generators might be applied, many of the aforementioned features and improvements can be readily implemented with the GenUI React components (see “10”) and the Python API (see “14”). In fact, the already presented extensions and the DrugEx interface are useful case studies that can be used as templates for integration of many other cheminformatics tools and de novo molecular generators. Therefore, we see GenUI as a flexible and scalable framework that can be used by organizations to quickly integrate tools and data the way it suits their needs the most. However, we would also like GenUI to become a new useful way to share the progress in the development of novel de novo drug design methods and other cheminformatics approaches in the public domain.

## Conclusions

We implemented a full stack solution for integration of de novo molecular generation techniques in a multidisciplinary work environment. The proposed GenUI software platform provides a GUI designed to be easily understood by experts outside the cheminformatics domain, but it also offers a feature-rich REST API for programmatic access and straightforward integration with automated processes. The presented solution also provides extensive Python and JavaScript extension APIs for easy integration of new molecular generators and other cheminformatics tools. We envision that the field of molecular generation will likely expand in the future and that an open source software platform such as this one is a crucial step towards more widespread adoption of novel algorithms in drug discovery and related research. We also believe that GenUI can facilitate more engagement between different groups of users and inspire new directions in the field of de novo drug design.

## Data Availability

The complete GenUI codebase and documentation is distributed under the MIT license and located in three repositories publicly accessible on GitHub: https://github.com/martin-sicho/genui (backend Python code); https://github.com/martin-sicho/genui-gui (frontend React application); https://github.com/martin-sicho/genui-docker (Docker files and deployment scripts). A reference application that was described in this manuscript can be deployed with Docker images that were uploaded to Docker Hub: https://hub.docker.com/u/sichom. However, the images can also be built with the available Docker files and scripts (archived at 10.5281/zenodo.4813625). The reference web application uses the following versions of the GenUI software: 0.0.0-alpha.1 for the frontend React application (archived at 10.5281/zenodo.4813608); 0.0.0.alpha1 for the backend Python application (archived at 10.5281/zenodo.4813586).
